# EVIDENCE-BASED STRATEGIES TO DELIVER AND PROMOTE AGE-FRIENDLY CARE IN HOSPITAL SETTINGS

**DOI:** 10.1093/geroni/igad104.0190

**Published:** 2023-12-21

**Authors:** Barbara King, Mary Hook, Blair Golden

## Abstract

The number of adults aged 65 and older will double by 2050. Older adults have more chronic conditions and complex care needs compared to younger adults. To support the health of older adults the Age Friendly Health Systems model was initiated in the US. Age Friendly Care is comprised of 4 core elements (called the 4 M’s framework), What Matters, Medication, Mentation and Mobility, aimed to ensure that older adults receive the best care possible and are not harmed by health care systems. This symposium will present several papers describing how components of age friendly care were implemented in hospital settings. Paper 1 will describe strategies used by acute care nursing staff to overcome challenges imposed by COVID to implement a mobility intervention to improve ambulation of older adult patients; Paper 2 will present psychometric findings on an instrument to measure a patient’s level of ambulation to promote mobility during a hospital stay; Paper 3 will describe a quality improvement initiative to implement the 4 M’s framework on in-patient medical units; and Paper 4 will provide insights on implementing the What Matters component of 4 M care and strategies for promoting patient goals and preferences for care delivery. Age-Friendly Health Systems have the potential to improve care delivery for older adults. The papers presented demonstrate it is feasible to implement 4 M’s framework in hospitals, but innovative strategies and reliable tools are needed.

